# MALDI-MS: a Rapid and Reliable Method for Drug-to-Antibody Ratio Determination of Antibody-Drug Conjugates

**DOI:** 10.29252/ibj.23.6.395

**Published:** 2019-11

**Authors:** Mehri Abedi, Reza Ahangari Cohan, Fereidoun Mahboudi, Mehdi Shafiee Ardestani, Fatemeh Davami

**Affiliations:** 1 Biotechnology Research Center, Pasteur Institute of Iran, Tehran, Iran;; 2 Department of Pilot Nanobiotechnology, New Technologies Research Group, Pasteur Institute of Iran, Tehran, Iran;; 3 Department of Radiopharmacy, Faculty of Pharmacy, Tehran University of Medical Sciences, Tehran, Iran

**Keywords:** Antibody-drug conjugate, Mass spectrometry, Trastuzumab

## Abstract

**Background:**

It is believed that the loading value of anticancer drug conjugated to the monoclonal antibody, called drug-to-antibody ratio (DAR), is the main quality feature of antibody-drug conjugates.

**Methods:**

In this study, matrix assisted laser desorption/ionization mass spectrometry was used to determine the average molecular weight of trastuzumab and its three conjugated forms. The differences in the measured masses for each conjugate and unconjugated trastuzumab were compared to the expected mass change through the conjugation of one mole of related drug-linker, in order to measure DAR.

**Results:**

There was a consistency between the loading results of mass spectrometry and the measurements of UV spectrometry in most cases. **Conclusion:** According to our findings, the MALDI-MS method for determining the loading values can be used rapidly and reliably to estimate the covalently bound drugs conjugated to antibodies when ESI-TOF-MS is unavailable.

## INTRODUCTION

Monoclonal antibodies have actively been used to deliver the anticancer drugs to the sites of tumors. These small anticancer drug molecules conjugate to the monoclonal antibody chemically or enzymatically, leading to the formation of antibody-drug conjugate (ADC). The loading value of a drug on the conjugate is defined as the average number of moles of that drug attached to a monoclonal antibody. The loading value is considered as the major quality feature of an ADC since it specifies the payload amount that reaches the tumor cells and can straightly alter safety and efficacy of the conjugate^[^^1^^-^^8^^]^. The UV/VIS spectroscopic analysis of the ADC is known to be the easiest procedure to determine this feature. The maximum absorbance values of the UV/VIS spectra of the drug and the antibody should be different to implement this procedure. The concentrations of mAb and drug can be calculated separately by solving two equations at the same time using the ADC’s measured absorbance and the mAb’s extinction coefficients at 280 nm and the drug at its λ_max_. Then the molar ratio can be determined, which refers to the moles of drug per mole of antibody. It is necessary to integrate the portion of the drug to the measured absorbance at 280 nm and any protein quota to the measured absorbance at the drug λ_max_^[^^9^^-^^12^^]^. The reliability of the spectroscopic method can also be confirmed by applying orthogonal techniques such as radiometric^[^^13^^]^ and chromatographic^[^^11^^]^ methods. 

According to the chemistry used for the drug-to-antibody conjugation, various methods have been introduced to determine the drug-to-antibody ratio (DAR). In the case of lysine amide conjugation, it would be difficult to separate conjugates by chromatography because of their high heterogeneity. Evidence has shown the application of mass spectrometry for the analysis of these ADCs^[^^14^^]^. UV MALDI-TOF method was one of the first instances of ADCs characterization by mass spectroscopy in the early 1990s in which a comparison was made between the mass spectra of intact conjugated mAbs and the related parent monoclonal antibodies. The results of this method were not desirable in terms of low mass accuracy for large molecules, and due to limited resolution, it could not supply resolution of various forms of ADCs with different drug loads. However, the mass change of the peak centroids was used to define the average DAR, and the peak configuration was applied to model the distribution^[^^15^^]^. LC-MS with electrospray ionization coupled to time-of-flight (TOF) or triple quadrupole mass detectors were used by previous investigations such as those focusing on the analyses of T-DM1 (trastuzumab-MCC-DM1) and thio-trastuzumab-DM^[^^16^^]^, huN901-SPP-DM^[^^17^^]^, and C242-DM4^[^^18^^]^. These techniques yield more stringent mass and resolution than can be gained using MALDI.

 The goal of this study was to compare the DAR values acquired from UV spectroscopy with the related values resulted from intact mass measurement by MALDI-TOF/TOF method. Actually, we attempted to show that in cases where ESI-TOF-MS is not available, intact mass measurement of conjugates by MALDI-TOF/TOF mass spectroscopy could be a reliable technique to calculate the DAR values of conjugates. For this purpose, three different linkers with different masses and length sizes (Table 1), including SMCC, SM(PEG)_2_, and SM(PEG)_12_, were applied to conjugate DM1 drug molecule to the trastuzumab antibody. 

After confirmation of conjugation reaction by SDS-PAGE and peptide mapping analysis, conjugates were subjected to UV spectroscopy and mass spectrometric analysis to calculate DAR of each conjugate. Comparison of the results indicated a logical correlation between the loading results obtained by mass spectrometry and the measurements acquired by UV spectrometry.

**Table 1:** Physical properties of used linkers

**Linker **	**Spacer arm ** **length (A˚)**	**Molecular ** **weight (Da)**
SMCC	8.3	334.32
SM(PEG)_2_	17.6	425.39
SM(PEG)_12_	53.4	865.92

## MATERIALS AND METHODS

**Drug, linkers, and antibody**

N2'-Deacetyl-N2'-(3-mercapto-1-oxopropyl)-mayta-nsine, also known as Mertansine or DM1, was from Carbosynth Limited, Berkshire, UK (FD33453). The succinimidyl-4-(N-maleimidomethyl)-cyclohexane-1-carboxylate (SMCC) linker was obtained from Sigma-Aldrich, Saint Louis, USA (M5525-100MG). Hydrophilic PEG linkers, SM(PEG)2 (succinimidyl-[(N-malei-midopropio-namido)-diethyleneglycol]) ester and SM(PEG)_12 _(succinimidyl-[(N-maleimido propionamido)-dodeca-ethyleneglycol]) ester were procured from Thermo Fisher Scientific, Illinois, USA (22102 and 22112). Trastuzumab and kadcyla^®^ were purchased from AryoGen Pharmed, Tehran, Iran (500015300006) and Genentech Roche, California, USA (980-946), respectively.

**Conjugates synthesis**

**Fig. 1:**
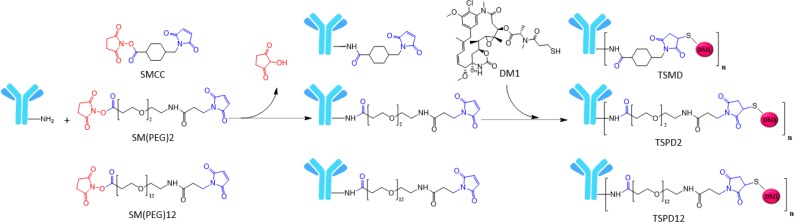
Two-step conjugation of DM1 drug to trastuzumab via three different linkers. The first step of the conjugation involves reacting the N-hydroxysuccinimide ester of each linker with available lysine residues on the antibody and formation of a stable amide bond with the primary amine of lysine residues. In the second step, the maleimide moiety of attached linkers reacts with sulfhydryl group on DM1 drug to form stable thioether bonds. The process resulted in the formation of a mixture of conjugates with diferent DARs

Conjugates were synthesized according to Steeves *et al.*^[^^19^^]^. The resulting conjugates bearing SMCC, SM(PEG)_2_, and SM(PEG)_12_ linkers were named as TSMD, TSPD_2_, and TSPD_12_, respectively. Physical properties of the mentioned linkers and conjugation steps are shown in Table 1 and Figure 1, respectively.

**Analytical methods**

***SDS-PAGE analysis***

The coupling of DM1 to light and heavy chains of antibody was further investigated using SDS-PAGE analysis according to the standard protocols. Each trastuzumab ADC was electrophoresed in both 12 and 12-16% gradient gels using PROTEAN^®^ II xi cell (Bio-Rad, Hercules, CA, USA) and visualized by Coomassie blue staining method. Data acquisition was done using GS-800™ calibrated densitometer and Quantity One Software (Bio-Rad). 

***Peptide mapping analysis***

Peptide mapping analysis was performed for trastuzumab and each trastuzumab-drug conjugate according to the USP compendium^[^^20^^]^. Briefly, 200 µL of each sample (10 mg/mL) was reduced using the denaturing buffer containing 10 ml of guanidine chloride (6 M), 1 mM of EDTA in Tris 0.25 M (pH 7.5 ± 0.1), and 20 µl of 0.5 M DTT at 37 °C for 30 min. 

Then 48 µl of iodoacetamide 0.5 M was added and incubated in the dark at room temperature for 30 min, and afterward, alkylation was stopped by adding 20 µl of 0.5 M DTT. Digestion buffer was introduced using 10 kDa Microcon^®^ Centrifugal Filter (Millipore, Massachusett, USA) to reach the final concentration of 1 mg/mL. Finally, 20 µl of trypsin (1 mg/mL) was added to 500 µl of each sample and incubated at 37 °C for 18 h. The digestion reaction was stopped by adding trifluoroacetic acid 10%. A C_18 _column (5 µm particle size, 4.6 mm ID × 15 cm length, Tosoh Bioscince, Japan) was equilibrated with 96% mobile phase A (0.1% TFA in water for injection) and 4% mobile phase B (0.1% TFA in acetonitrile) at 40 °C over 30 min and a flow rate of 1 mL/min. Following the sample injection (100 µl), 4% mobile phase B was injected to the column for 5 min and increased to 30% over 75 min. Again, the mobile phase B was further increased to 40% over 30 min and finally reached 100% in 5 min. Solvent composition was then returned to 4% in 5 min and held for 10 min. Detection was done at 214 nm (peptide bond absorbance wavelength) using UV detector S2600 Smartline (Knauer, Berlin, Germany).

***UV spectroscopy***

UV spectra were individually acquired from kadcyla^®^, trastuzumab, and each trastuzumab ADCs (1 mg/mL in PBS pH 6.5) in the wavelength range of 200 to 360 nm using Epoch Microplate Spectro-photometer (Biotek, Winooski, USA) in a UV-Star® 96-Well Microplate (Greiner Bio-One GmbH, Germany). The UV spectra of DM1 were also obtained to show its maximum absorbance at 252 nm. The data were acquired by Gen5^™^ software version 2.00.18, and spectra were plotted using GraphPad Prism version 6 software.

***Extinction coefficient calculations***

In order to calculate DAR of each conjugate, extinction coefficient values of trastuzumab and DM1 at 252 and 280 nm wavelengths were calculated as follow^[^^21^^-^^23^^]^. For trastuzumab, 0.1% solution (1 mg in 1 mL of water for injection) was prepared. Then two-fold serial dilutions were prepared in triplicate in a 96-well UV-Star^®^microplate (Greiner Bio-One GmbH). Absorbance was measured at 252 and 280 nm wavelengths using Epoch Microplate Spectro-photometer (Biotek), and standard curves were plotted. Line equation of each standard curve (equation 1) was used to calculate the extinction coefficient values using Beer-Lambert equation (equation 2). 

(1)                   Y=aX+b

(1a)                    $a=\frac{Y-b}{X}$

 Where Y and X are the absorbance and concentration values, respectively, and a is the slope of the line equation.

(2)                    A=ƐCL

 Where Ɛ is the molar extinction coefficient, C is concentration of the dissolved absorbing molecule, and L is the path length that light has to pass through the material. In order to determine the molar extinction coefficient of the trastuzumab, equation 2 rearranged as below:

(2a)                    $ƐL=\frac{A}{C}$

 An equality was set up (eq 1a = eq 2a) and solved for extinction coefficient (equation 3):

(3)                    $Ɛ=\frac{a}{L}$

 Where the value of L in UV-Star^®^microplate is 0.56 cm, extinction coefficient is the extinction coefficient_0.1% __solution_ of the trastuzumab, and once it is calculated, the value is used to solve for extinction coefficient molar in equation 4.

(4)                    Ɛ Molar=Ɛ 0.1% solution ×MW Trastuzumab

Where the molecular weight of trastuzumab is 145423 g/mol.

 In order to calculate the extinction coefficient values of DM1 at 252 and 280 nm wavelengths, a stock solution of DM1 (2.7 mM in dimethylacetamide) was prepared. The rest of the steps are the same as the steps listed for trastuzumab, except for equation 4, which is as follows.

(5)                    Ɛ Molar=Ɛ milli molar ×1000

***DAR calculation by UV spectrometry***

DAR was measured according to Zhao *et al.*^[^^24^^]^. Absorbance of each trastuzumab ADC (1 mg/mL in PBS, pH 6.5) was individually measured at 280 and 252 nm wavelengths using Epoch Microplate Spectrophotometer (Biotek) in UV-Star^®^ 96-Well Microplate (Greiner Bio-One GmbH), and moles of DM1 per mole of trastuzumab were calculated using determined extinction coefficients and equation 6. Kadcyla^®^, as a known standar, was used to validate the measurement.

(6)                   DAR= $\frac{\left({ɛ\mathrm{Ab}}_{252}–{R\times ɛ\mathrm{Ab}}_{280}\right)}{\left({R\times ɛD}_{280}-{ɛD}_{252}\right)}$

Where ɛAb_252_ and ɛAb_280 _are extinction coefficients of trastuzumab at 252 and 280 nm, respectively; R is the ratio of absorbance of 252 nm to 280 nm of each conjugate; and ɛD_280 _and ɛD_252 _are extinction coefficients of DM1 at 280 and 252 nm, respectively.

***DAR calculation by MALDI-TOF/TOF mass spectrometry***

Matrix-assisted laser desorption/ionization mass spectrometry (Applied Biosystems 4800 MALDI-TOF/TOF, Foster City, CA, USA) was employed to determine the intact mass of kadcyla^®^, trastuzumab, and each of the trastuzumab ADCs. Before the test, the samples (at least 1.3 mg/mL) were desalted by C18 Zip-Tip reverse-phase chromatography pipette tip (Millipore, Burlington, Massachusetts, USA) according to the manufacturer’s instructions. The samples were then mixed with a matrix solution (1:1 v/v) of sinapinic acid in 50% ACN containing 0.1% TFA, spotted, air dried and analyzed with a MALDI-TOF/TOF mass spectrometer, operated in high linear positive mode. Finally, the data were processed using Data Explorer software version 4.0 from the supplier (Applied Biosystems). Using the MALDI molecular weights of each conjugate, the unconjugated antibody, and the substitution of drug and the molecular weights of linkers, as well as the DM1:trastuzumab molar ratios of each trastuzumab ADC were calculated through the following equation^[^^25^^-^^27^^]^.

 (7)                    $\frac{\mathrm{DM1}}{\mathrm{Trastuzumab}}$ = MWConjugate-MWTrastuzumabMW DM1+MWLinker-115

Where MW_Conjugate_, MW_Trastuzumab_, MW_DM1, _and MW_Linker_ are the molecular weights of each trastuzumab conjugate, trastuzumab, DM1, and linker, respectively, and 115 is the molecular weight of the N-Hydroxysuccinimide leaving group of each linker after mAb coupling.

## RESULTS

**Molecular weight analysis**

**Fig. 2:**
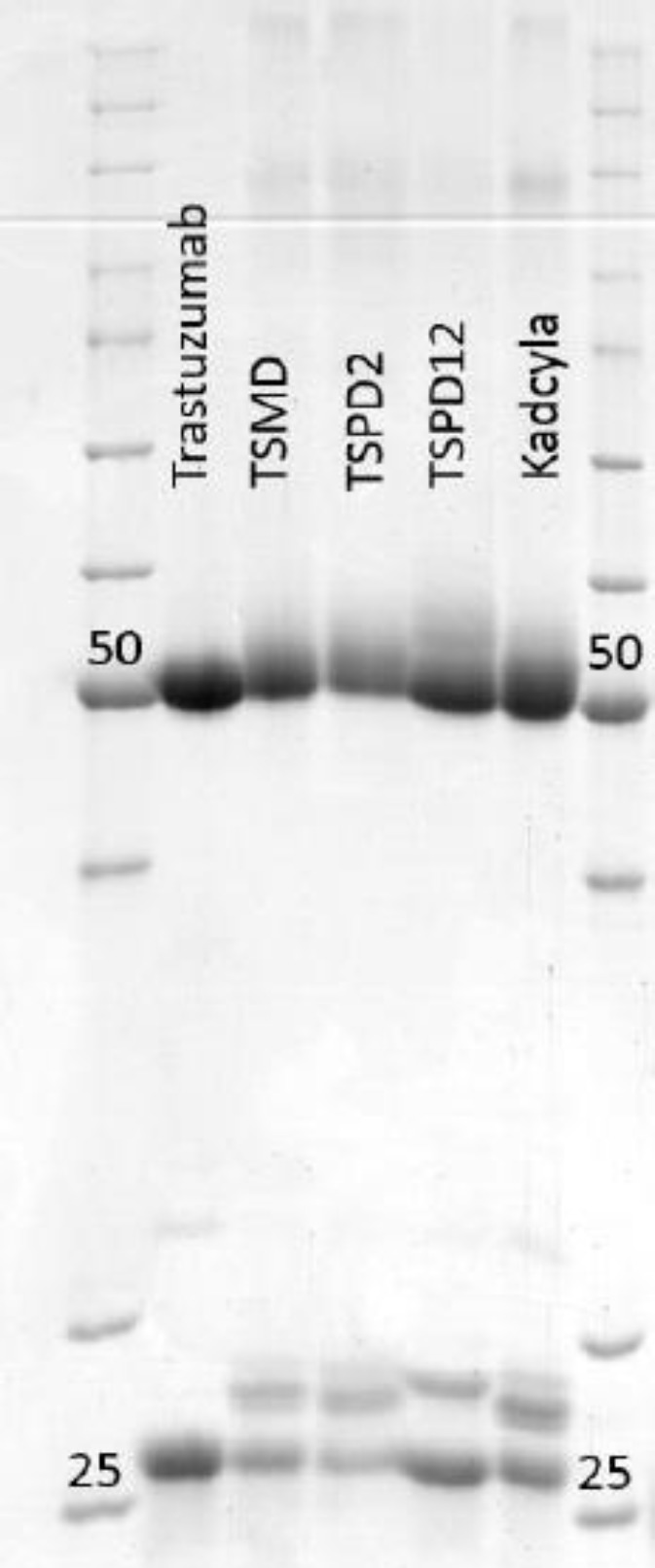
SDS-PAGE analysis of conjugates on 12% poly-acrylamide, trastuzumab, and kadcyla^®^, which were used as negative and positive controls, respectively

Kadcyla^®^, trastuzumab, and the conjugates were subjected to 12% sodium dodecyl polyacrylamide gel electrophoresis (Fig. 2). In 12% gel, the unconjugated (25 kDa) and conjugated light chains (heavier than 25 kDa) were clearly defined as individual bands, along with negligible resolution of unconjugated heavy chains. The test was repeated several times on 12-16% gradient gels to increase the resolution of unconjugated (50 kDa) and smear shape conjugated forms of heavy chains (data not shown).

**Analysis of tryptic digests**

**Fig. 3:**
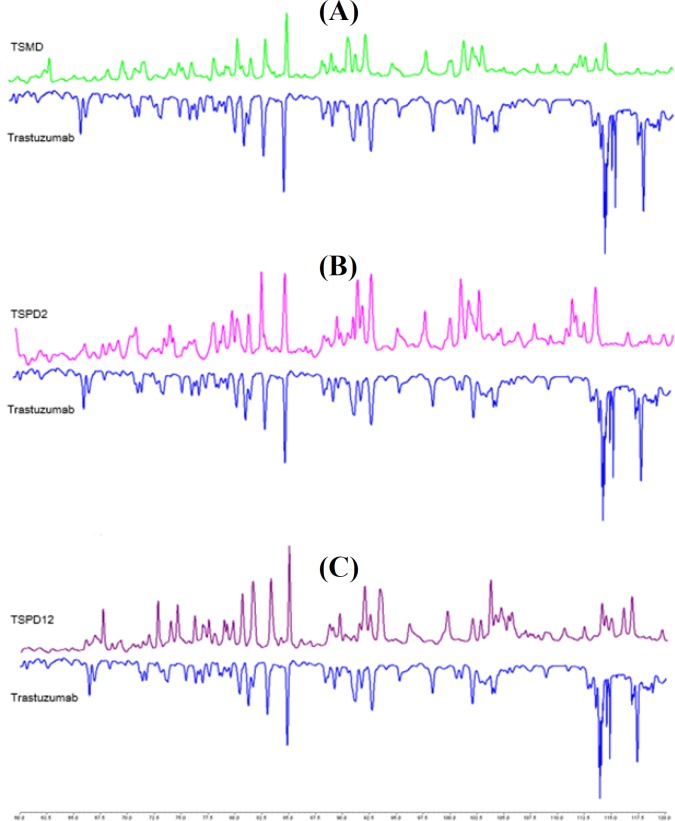
Peptide mapping of conjugates. Comparison of mirror plots of tryptic digests of trastuzumab with (A) TSMD, (B) TSPD_2_, and (C) TSPD_12_ at 214 nm

Peptide maps of the trastuzumab and conjugates were compared as a further confirmatory test. Comparison mirror plots of tryptic digests of trastuzumab and each conjugate at 214 nm showed observable changes related to conjugation as linker modified lysines were not cleavable by the enzyme (Fig. 3). 

**Conjugation verification by UV spectroscopy**

**Fig. 4:**
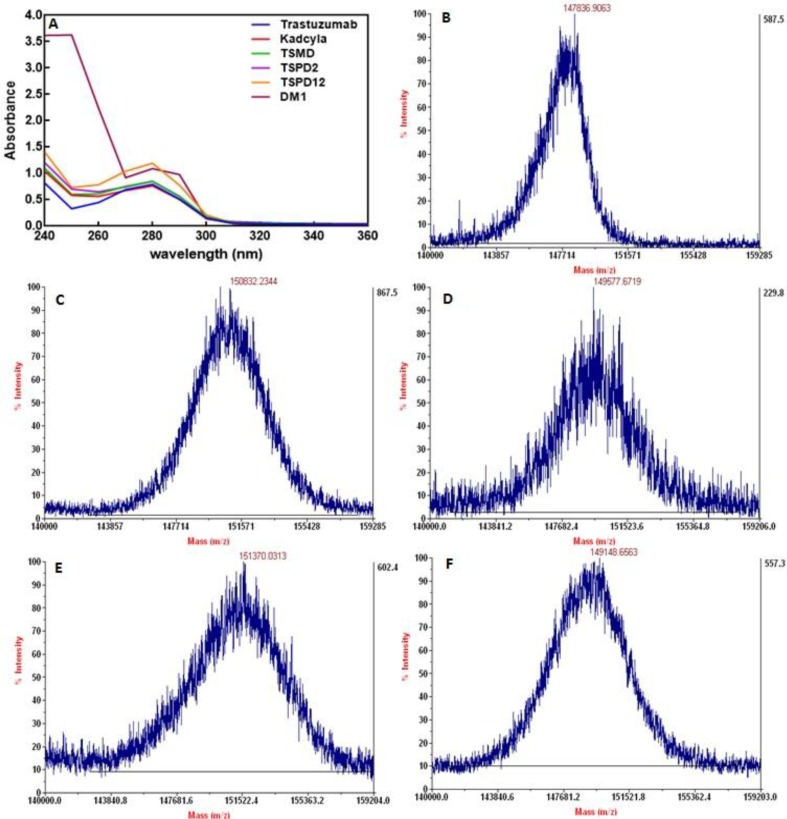
UV spectroscopy and mass spectrometry of conjugates. (A) UV spectra analysis revealed an increase in absorbance at the wavelength of 252 nm of conjugates. MADLI -TOF/TOF intact mass spectrum of (B) Trastuzumab, (C) Kadcyla®, (D) TSMD, (E) TSPD_2_, and (F) TSPD_12_

UV spectra of Kadcyla^®^, trastuzumab, and the conjugates in the wavelength range of 240 to 360 nm revealed an absorption increasement in all conjugates in comparison to the unconjugated antibody spectrum at the wavelength of 252 nm, which is the lambda max of the drug (Fig. 4A). 

**DAR values**

The average number of conjugated drugs per antibody was calculated using determined molar extinction coefficients (ɛ_252nm_ = 25600 M^-1^cm^-1^ and ɛ_280nm _= 4410 M^-1^cm^-1^ for DM1, ɛ_252nm _= 74311 M^-1^cm^-1^ and ɛ_280nm_ = 209409 M^-1^cm^-1^ for trastuzumab). Calculated DAR value for Kadcyla® was very close to the previously reported value (3.5), which confirmed the accuracy of the measurement. DARs for conjugates are shown in Table 2.

**Intact MS analysis**

MALDI-TOF-TOF of Kadcyla®, trastuzumab, and its conjugates demonstrated an increase in the intact mass of all conjugates compared with trastuzumab without any additional peaks (Fig. 4B-4F). The DAR values obtained by mass spectrometry are shown in Table 2.

## DISCUSSION

ADCs are now of considerable interest and are recommended for the treatment of cancers. It is believed that the loading value of anticancer drug conjugated to the monoclonal antibody, called DAR, is the main quality feature of ADCs. Various methods have been introduced to determine the DAR according to the chemistry used for the drug-to-antibody conjugation. Because of the importance of DAR value and the lack of easy access to ESI-TOF-MS in our country, we applied two methods (UV spectrometry and MALDI TOF/TOF mass spectrometry) in parallel to calculate the DAR value of three synthesized trastuzumab-DM1 conjugates. Conjugation reaction was confirmed by SDS-PAGE and peptide mapping analysis, which both verified the successful linkage of DM1 molecules to the antibody via the linkers.

SDS-PAGE has been found to be a suitable qualitative method for resolving drug-coupled antibody chains from the unconjugated ones, only based on molecular weight difference. In this regard, studies have employed SDS-PAGE for this purpose^[^^28^^, ^^29^^]^. Our analysis also distinguished between the chains coupled to the drug and the unconjugated ones. The results revealed that both light and heavy chains were modified with the drug and, as expected, the conjugated chains showed a higher molecular weight in comparison with uncoupled chains. However, a lower resolution was observed for the heavy chains, as compared to the light ones. Gradient gel electrophoresis using higher polyacrylamide concentrations enhanced the resolution of band separation, especially for light chains. This improvement was not significant for the heavy chains, probability due to glycosylation phenomena, which led to a smear-shape of bands on the polyacrylamide gel. Based on the net mass values, SM(PEG)_12_ linker is heavier than SM(PEG)_2_ and SMCC linkers. SDS-PAGE analysis also revealed that conjugated light and heavy chains of conjugate bearing SM(PEG)_12_ linker were slightly heavier than light and heavy chains of conjugates containing SM(PEG)_2_ and SMCC linkers.

**Table 2:** Masses and drug-to-antibody ratio of trastuzumab-DM1 conjugates

**Antibody/** **conjugate**	**Measured ** **mass (Da)**	**Mass shift:** **conjugate mass- trastuzumab mass (Da)**	**Mass addition per each conjugated drug linker (Da)**	**Drug-to-antibody ratio (DAR)**	
				**MS**	**UV**
Trastuzumab	147,836.9	---	----	---	---
Kadcyla^®^	150,832.2	2995.3	957.4	3.1	3.3
TSMD	149,577.7	1740.8	957.4	1.8	2.7
TSPD_2_	151,370.0	3533.1	1,048.4	3.4	4
TSPD_12_	149,148.6	1311.7	1,488.7	0.9	2

DAR for each conjugate was calculated by two methods, the mass change on conjugation, measured by MS, and UV absorbance measured by UV spectroscopy of conjugates at 252 and 280 nm.

Peptide mapping is a robust technique for protein modification tracing such as conjugation in which protease activity is affected by conjugation. In the case of lysine conjugations, trypsin will not cleave at lysines upon drug attachment, leading to a different peptide map. Product identity will be determined by comparing exclusive chromatographic profiles obtained from enzymatic digests of an ADC^[^^14^^,^^17^^]^. Altered peptide mapping patterns of our synthesized conjugates in comparison to trastuzumab verified the modification of lysine residues related to the conjugation of linkers to antibody via lysine residues.

UV spectroscopy revealed an increase in absorbance at 252 nm wavelength in all ADCs, which could be interpreted by the attachment of DM1 molecules to the antibody because DM1 has a maximum absorbance at this wavelength. DAR of each of the conjugates was then calculated through absorbance measurements of each conjugate at lambda max wavelengths of the drug and antibody. UV spectroscopy was also popularly used for the conjugation assessment either in ADCs synthesis or other chemical reactions^ [^^9^^-^^11^^,^^30^^-^^34^^]^. 

MALDI-MS is usually used to analyze molecular weight directly and measures the substituent-to-MAb (S:A) ratio clearly in real time; it has already been used for conjugation of small molecules to antibodies^[^^25^^-^^27^^]^. Our MADLI-TOF/TOF intact mass spectrum analysis determined specified mass to charge ratios for each ADC. Successful conjugation was confirmed as centroid data revealed expected positive mass shifts of drug-loaded antibodies in comparison to unconjugated one. The number of DM1 conjugated to trastuzumab was calculated from the differences in the measured masses for each conjugate and unconjugated trastuzumab relative to the expected mass change upon the conjugation of one mole of related drug-linker. The acquired values indicated a consonant relationship with DAR values obtained by UV spectrometry for all ADCs. There was also a difference between the MALDI-MS-derived and UV spectrometry-derived estimations; this difference could be associated with the combined variations in both assay methods. 

Our results are in accordance with Siegel *et al.*^[^^15^^]^ study, in which the loading values of metal chelators and anticancer drugs conjugated to monoclonal antibodies were measured and compared using UV and mass spectroscopic assays. In another study by Safavy *et al.*^[^^25^^]^, the progress and the extent of the PTX-C225 conjugation reaction were followed by MALDI-MS. According to their results, MALDI-MS analyzed and optimized the conditions for PTX-C225 conjugation clearly and efficiently; it measured the PTX:C225 ratios directly.

According to our findings and other studies in this field, it could be concluded that the MALDI-MS method could be appropriate for calculating the DAR rapidly and reliably; besides, it could be used to estimate the covalently bound drugs conjugated to antibodies when ESI-TOF-MS is not available. 
